# Carelessness or Ignorance, Which?

**Published:** 1880-08

**Authors:** 


### EDITORIAL.

### CARELESSNESS OR IGNORANCE, WHICH?

How can the operations of the average dentist be improved? It may be answered thus; either by greater care, or an increase of knowledge and skill, the improvement in one or the other or both of these respects, of those now in the practice of dentistry, is a subject of no mean importance; it is one which in some respects at least is equal to the education of the student. Why is it that there are so many failures in the ordinary practice of the common dentists, and even of those a grade or two above the average, and occasionally with those who are regarded as among the best.

A few cases may illustrate what is here meant A little while ago, a first inferior molar of the right side was presented to the writer of this for an opinion and treatment if necessary, a rather large but not deep cavity on the buccal surface, extending fully half a line below the margin of the gum, had been filled within a year with soft foil,—it was very soft—by a man extensively known as a dentist, and of more than forty years' experience, and one who I know has had large opportunity for gaining professional knowledge. The gum in contact with this filling was highly inflamed, quite red, swollen and very sensitive to the touch, upon examination, the filling? stuffing was found protruding from the edge of the cervical wall of the cavity, fully half a line, with its rough, ragged edge presented to the inner border of the gum and holding in contact with the latter, the vitiated, putried, poisonous matter that had accumulated there, the disease was extending to the gum of the adjoining teeth, the dental periosteum was also somewhat affected and doubtless would soon have been seriously so. Now, how came this about? Presumably, largely from *sheer, criminal carelessness*, it may in part however, be due to a doltish ignorance that has closed its eyes to the light and progress of the last twenty-five years.

Another case, a young lady of eighteen called upon a practitioner of over twenty-five years' experience, to fill a small cavity on the anterior proximal surface of the left superior incisor, the cavity was formed cone-shaped with base outward, and filled—stuffed; within a few weeks the filling was out, then the cavity was cut larger, the shape not much changed and filled again, and in a short time with the same result as before, then another attempt was made and by the excavation this time, the pulp was exposed, then attempted to cover and fill, the pulp after some aching, became devitalized and the filling was lost the third time and with it went the patient's patience, when I saw it, an abscess was forming.

This is a clear case of "*Didn't know how.*" And why did he not know? Simply because he would not, though his boast was that he had twenty-five years' experience. Well what of it? That is about all he did have. It is however an abuse of the word experience to use it in such a connection, it was in reality twenty-five years and more of wretched bungling, based on egotism and ignorance.

Another case, a dentist of about thirty years' *experience*—"*sperience*," had not long since, presented for the exercise of his skill, a case of diseased gums, the gums about all the teeth,—the set was complete with the loss of but two or three teeth,—were inflamed, swollen, sensitive and somewhat painful, there was little or no calculus apparent upon the teeth, and so the treatment adopted and continued for two or three weeks, was the application of tannin, various solutions, dilute carbolic acid, iodine, aromatic sulphuric acid, &c., &c., none nor all of which made much impression so far as restoration to health was concerned, the case was thought to be a very obstinate one by the dentist, the patient was most of the time "bad and then worse again." An inspection of this case by one more searching, found upon the decks of all the teeth, beneath the free margins of the gum a little ring of hard, dark calculus completely hidden by the gum, the complete removal of this without anything further, resulted within a few days in a great change for the better, and in a short time the gums were in a very good condition.

This was a case of carelessness, the results of which was just as bad as the most palpable ignorance, such inefficiency or neglect as shown in these cases, should if habitual, bar anyone from practice, it should be sufficient ground upon which to cancel a diploma or license.

